# Evaluation of the endocranial anatomy of the early Paleogene north African gavialoid crocodylian *Argochampsa krebsi* and evolutionary implications for adaptation to salinity tolerance in marine crocodyliforms

**DOI:** 10.1111/joa.14213

**Published:** 2025-01-15

**Authors:** Carly C. Pligersdorffer, Paul M. J. Burke, Philip D. Mannion

**Affiliations:** ^1^ Department of Earth Sciences University College London London UK; ^2^ School of GeoSciences, Grant Institute University of Edinburgh Edinburgh UK

**Keywords:** crocodylians, Gavialoidea, neuroanatomy, salt glands, transoceanic dispersal

## Abstract

*Argochampsa krebsi* is a gavialoid crocodylian from the early Paleogene of North Africa. Based on its recovered phylogenetic relationship with South American species, it has been inferred to have been capable of transoceanic dispersal, but potential anatomical correlates for a marine lifestyle have yet to be identified. Based on CT scans of a mostly complete and well‐preserved skull, we reconstruct the endocranial anatomy of *Argochampsa* and compare it to that of other gavialoids. We demonstrate that *Argochampsa* possesses concave depressions on the internal surface of the prefrontals and lacrimals, which have been inferred to represent osteological correlates for salt glands in unequivocally marine metriorhynchoid thalattosuchian crocodyliforms. The presence of these salt glands suggests that *Argochampsa* likely frequented pelagic environments and provides additional support for the capability of transoceanic dispersal within Gavialoidea. We also newly interpret osteological correlates for salt glands in the Miocene north African gavialoid *Sutekhsuchus dowsoni*, providing further support that saltwater tolerance was widespread and possibly ancestral in Gavialoidea, given that they have been previously reported in the Late Cretaceous–early Paleogene species *Eosuchus lerichei* and *Portugalosuchus azenhae*. In addition to these gavialoids, as well as metriorhynchids, we also identify these osteological salt gland correlates in the Paleocene northwest African dyrosaurid *Rhabdognathus aslerensis*, which represents another crocodyliform lineage thought to be capable of transoceanic dispersal. Given that dyrosaurids, gavialoids, and metriorhynchoids are distantly related lineages, the evolution of salt glands is likely a convergent ecological adaptation to the occupation of pelagic environments. Nevertheless, we demonstrate limited evaluation of the presence of these osteological correlates across Crocodyliformes, including within most extant species, such that it remains possible that they are much more widespread.

## Abstract 2

We reconstructed the endocranial anatomy of Argochampsa krebsi, a fossil gavialoid crocodylian from the early Paleogene of North Africa, identifying potential osteological correlates for salt glands. The salt gland osteological correlates found in Argochampsa krebsi are the same as those observed in other crocodyliforms. Our results provide evidence for the transoceanic dispersal of Gavialoidea and suggests convergent evolution of salt glands in different crocodyliform lineages.

## 1 INTRODUCTION

Whereas Crocodylia has a circumtropical distribution (Grigg & Kirshner, 2015), the subclade Gavialoidea, represented only by *Gavialis gangeticus* and *Tomistoma schlegelii*, is restricted to southern Asia (Shaney et al., 2019; Stevenson & Whitaker, 2010). However, the fossil record shows that extinct crocodylians had a much more global distribution. This includes gavialoids, which were not only more widespread across Asia, but extended into Europe, North America, South America, Africa, and Australasia (e.g. Brochu, 2006; Iijima et al., 2022; Jouve et al., 2008; Nicholl et al., 2020; Piras et al., 2007; Ristevski et al., 2021; Salas‐Gismondi et al., 2022; Vélez‐Juarbe et al., 2007). Note that here we follow topologies based on analyses of molecular, combined, and recent morphology‐only data matrics, in which *Gavialis* and closely related fossil species are recovered in a clade with *Tomistoma* (e.g. Iijima & Kobayashi, 2019; Lee & Yates, 2018; Pan et al., 2021; Rio & Mannion, 2021; Ristevski et al., 2022; Salas‐Gismondi et al., 2022), rather than some morphology‐based analyses in which *Gavialis* is placed as a distant lineage (e.g. Brochu, 2004, 2007; Jouve, 2016; Piras et al., 2007). This cosmopolitan distribution implies that gavialoids underwent transoceanic dispersal (e.g. Brochu, 2006; Buffetaut, 1982; Burke et al., 2024a, 2024b; Groh et al., 2023; Hua & Jouve, 2004; Jouve et al., 2008; Rio & Mannion, 2021; Salas‐Gismondi et al., 2019), but the two extant species are restricted to freshwater environments and are essentially saltwater intolerant (Taplin & Grigg, 1989). As such, most authors consider it likely that saltwater tolerance was ancestral for gavialoids and lost in the extant species (e.g. Delfino et al., 2005; Vélez‐Juarbe et al., 2007). Recently, Burke et al. (2024a) presented some of the first anatomical evidence to support the capability of transoceanic dispersal in fossil gavialoids. These authors interpreted the presence of salt glands based on internal features of the skull, revealed through computed tomography. Thus far, only a small number of gavialoid species have been evaluated in this way, limiting our ability to determine how widespread this capability was across Gavialoidea. Furthermore, the same features found on the internal surface of the skull have been demonstrated to be osteological correlates of salt glands in metriorhynchoid thalattosuchians (Cowgill et al., 2023).

One extinct gavialoid thought to be part of a lineage that was capable of transoceanic dispersal is *Argochampsa krebsi* (Hua & Jouve, 2004). This species is known from several specimens from the Couche IIb level of the phosphate series of the Ouled Abdoun Basin, in Khouribga Province, Morocco (Hua & Jouve, 2004; Jouve et al., 2006). This stratigraphic unit is dated to the Paleocene and can be approximately constrained to the upper Danian to lower Thanetian (Kocsis et al., 2014; Yans et al., 2014). Given that this is a marine deposit and that *Argochampsa krebsi* was recovered as an early branching member of a gavialoid clade that includes South American species and *Gavialis gangeticus*, Hua and Jouve (2004) proposed that this African taxon was capable of transoceanic dispersal. Subsequent phylogenetic analyses have continued to support a close relationship between *Argochampsa* and a clade composed of South American gavialoids and *Gavialis*, with potentially several transoceanic dispersal events required to explain the recovered topological arrangement (Burke et al., 2024a; Rio & Mannion, 2021; Salas‐Gismondi et al., 2016, 2019; Vélez‐Juarbe et al., 2007). However, anatomical features supporting marine capabilities have yet to be identified in *Argochampsa* (Hua & Jouve, 2004; Jouve et al., 2006).

**FIGURE 1:**
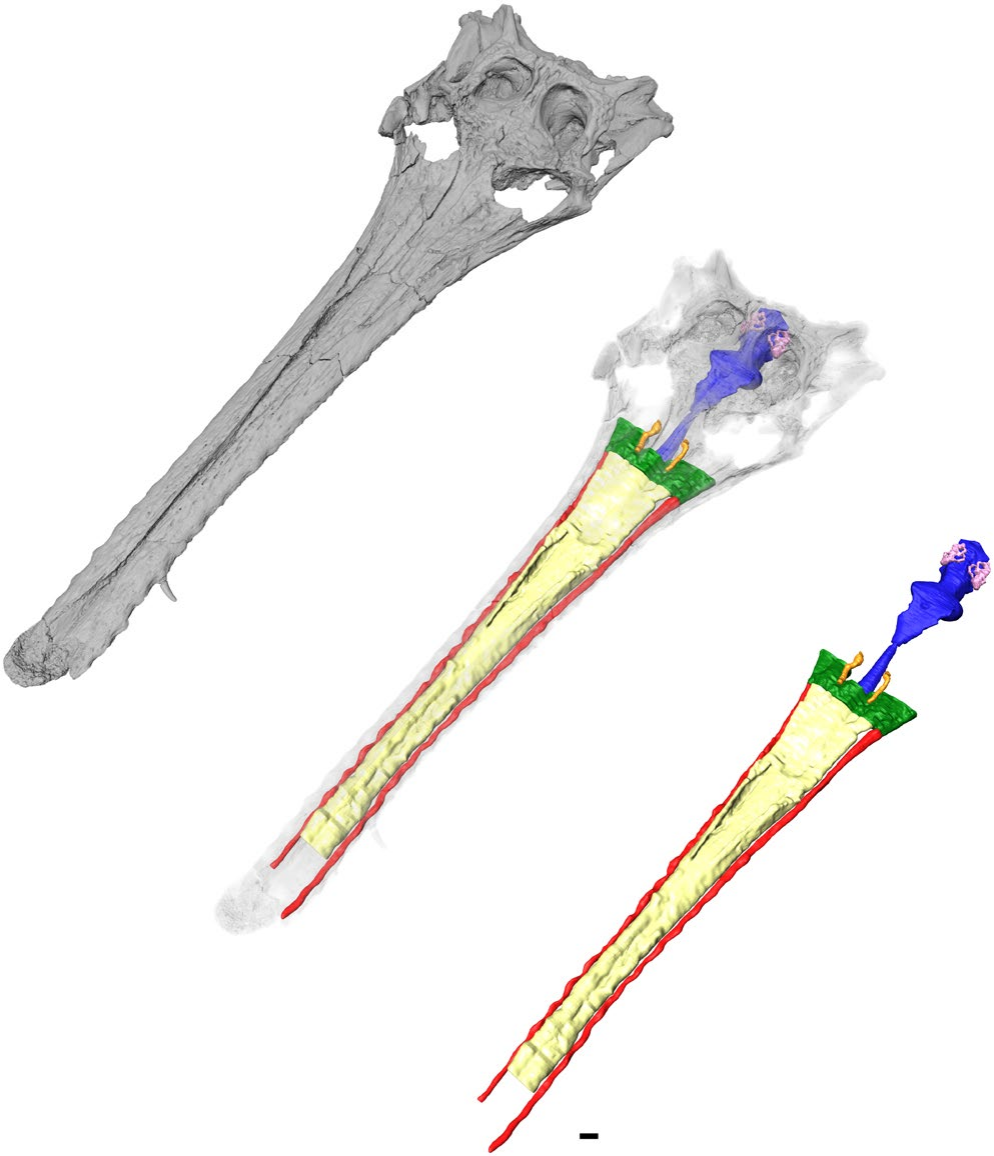
The skull and endocranial anatomy of *Argochampsa krebsi* (NHMUK PV R36872) in left dorsolateral view. Yellow, nasal cavity, red, neurovascular canals, orange, nasolacrimal ducts, green, olfactory region, blue, brain endocast, pink, endosseous labyrinth. Scale bar, 10 mm.

To overcome this problem, here we present a reconstruction of the endocranial anatomy of *Argochampsa krebsi* based on a previously undescribed specimen, using computed tomography to identify potential anatomical correlates for marine capability (Figure 1). We make anatomical comparisons with other gavialoids, as well as consider the evolution of adaptation to salinity tolerance, and thus capability of transoceanic dispersal, in *Argochampsa krebsi* and other marine crocodyliforms.

## 2 MATERIALS AND METHODS

### 2.1 Specimen provenance and stratigraphic age

The endocranial anatomy of *Argochampsa krebsi* was reconstructed from NHMUK PV R36872, which is a mostly complete and overall well‐preserved skull. This specimen was collected from the lower Ypresian (lowermost Eocene) Couche I level of the phosphate series of the Oulad Abdoun Basin in Khouribga Province, Morocco (D. Ward pers. comm., 2024). Its stratigraphic placement is supported by the surrounding matrix, which includes the selachian species *Abdounia beaugei* (Arambourg, 1935) (D. Ward pers. comm. 2024), which first appears in the Ypresian (Arambourg, 1952).

### 2.2 CT‐scan reconstruction

NHMUK PV R36872 was characterised at the NHMUK with X‐ray micro‐computed tomography using a Nixon Metrology XT H 225 system (Nikon Metrology, Leuven, Belgium). Acquisition of the skull was implemented in two parts, with a voltage of 210 kV and a current of 152 μA, resulting in a reconstructed isotropic voxel size of 90.341 μm^3^ and 2141 slices. NHMUK PV R36872 is dorsoventrally crushed near the prefrontal bones, resulting in the separation and displacement of the olfactory region from the rest of the endocast. Due to the poor preservation of the specimen in this region, the hindbrain and cranial nerves could not be reconstructed. This displacement was rectified in Avizo using the ‘Transform Editor’ which allowed the reconnection of the olfactory region with the rest of the endocast.

The 3D models of the endocranial structures were segmented manually in Avizo v. 9.7 (FEI Visualization Science Group; https://www.thermofisher.com), smoothed in MeshMixer (Autodesk; https://www.meshmixer.com), and rendered in Inkscape (Inkscape Project; https://inkscape.org).

Morphometric data were obtained from the endocast and endosseous labyrinth of NHMUK PV R36872 using the ‘Measurement’ tool in Avizo (Table 1). Following Pierce et al. (2017), the raw morphometric data were converted into ratios in order to interpret the relative proportions of the olfactory tract, cerebrum, pituitary fossa, and the endosseous labyrinth (Table 2). Comparative specimens used in this study are presented in Table 3.

**TABLE 1:** Measurements of the endocasts and labyrinths of crocodylian taxa.

Measurements (mm)	*Argochampsa krebsi* (NHMUK PV R36872) this study	*Gavialis gangeticus* (FLMNH UF118998) Burke and Mannion (2023)	*Gavialis gangeticus* (UMZC R5792) Pierce et al. (2017)	*Tomistoma schlegelii* (TMM M6342) Burke and Mannion (2023)	*Sutekhsuchus dowsoni* (NHMUK PV R4769) Burke and Mannion (2023)	*Eosuchus lerichei* (IRSNB R49) Burke et al. (2024a)
Skull width at cerebrum (b/w postorbitals)	83	135	168	68	109	95
Cephalic flexure angle	117	155	150	134	143	167
Pontine flexure angle	158	119	154	134	149	170
Endocast length	82	120	146	97	146	115
Olfactory tract length	28	49	55	47	66	52
Cerebrum width	23	28	32	27	30	26
Pituitary width	7	7	6	5	9	?
Pituitary height	5	9	9	8	9	?
Pituitary length	8	16	11	14	17	?
Maximum width of labyrinth	12	17	21	18	19	17
Maximum height of labyrinth	9	19	21	17	15	17
Endosseous cochlea length	4	10	9	10	11	?
Anterior semi‐circular canal area	44	20	36	17	21	43
Posterior semi‐circular canal area	10	6	15	5	10	9
Lateral semi‐circular canal area	5	10	22	4	?	12

**TABLE 2:** Ratios of endocast and labyrinth proportions of crocodylian taxa.

Measurements (mm)	*Argochampsa krebsi* (NHMUK PV R36872) this study	*Gavialis gangeticus* (FLMNH UF118998) Burke and Mannion (2023)	*Gavialis gangeticus* (UMZC R5792) Pierce et al. (2017)	*Tomistoma schlegelii* (TMM M6342) Burke and Mannion (2023)	*Sutekhsuchus dowsoni* (NHMUK PV R4769) Burke and Mannion (2023)	*Eosuchus lerichei* (IRSNB R49) Burke et al. (2024a)
Cerebrum width: Skull width	0.28	0.21	0.19	0.39	0.27	0.27
Cerebrum width: Endocast length	0.28	0.23	0.22	0.28	0.21	0.23
Olfactory tract length: Endocast length	0.35	0.41	0.38	0.48	0.45	0.45
Pituitary width: Pituitary height	1.36	0.78	0.67	0.63	1.00	?
Pituitary width: Pituitary length	0.91	0.44	0.55	0.36	0.53	?
Pituitary length: (Endocast‐Olfactory tract length)	0.15	0.23	0.12	0.28	0.21	?
Labyrinth width: Labyrinth height	1.38	0.89	1.00	0.94	0.79	1.00
Cochlear duct length: Labyrinth height	0.40	0.53	0.43	0.56	0.58	?
Anterior semi‐circular canal area: Posterior semi‐circular canal area	4.19	3.33	2.40	3.40	2.10	4.78
Anterior semi‐circular canal area: Lateral semi‐circular canal area	8.25	2.00	1.64	4.25	?	3.58
Posterior semi‐circular canal area: Lateral semi‐circular canal area	1.97	0.60	0.68	1.25	?	0.75

**TABLE 3:** Specimens used for comparative anatomical analysis.

Species	Specimen number	Reference
*Argochampsa krebsi*	NHMUK PV R36872	This study
*Argochampsa krebsi*	OCP DEK‐GE 1201	Hua & Jouve, 2004
*Eosuchus lerichei*	IRSNB R49	Burke et al., 2024a
*Gavialis gangeticus*	FLMNH UF118998	Burke & Mannion, 2023
*Gavialis gangeticus*	UMZC R5792	Pierce et al., 2017
*Ocepesuchus eoafricanus*	OCP DEK‐GE 45	Jouve et al., 2008
*Sutekhsuchus dowsoni*	NHMUK PV R4769	Burke & Mannion, 2023
*Tomistoma schlegelii*	TMM M6342	Burke & Mannion, 2023

Institutional abbreviations. IRSNB, Institut Royal des Sciences Naturelles de Belgique, Brussels, Belgium; FLMNH, Florida Museum of Natural History, Gainesville, Florida, USA; MHNT, Muséum d'Histoire Naturelle de Toulouse, Toulouse, France; NHMUK, Natural History Museum, London, United Kingdom; OCP, Office Chérifien des Phosphates, Casablanca, Morocco; TMM, Texas Memorial Museum, Austin, Texas, USA; UMZC, University Museum of Zoology, Cambridge, UK.

## 3 TAXONOMIC IDENTIFICATION

NHMUK PV R36872 was accessioned as a specimen of either *Argochampsa krebsi* or *Ocepesuchus eoafricanus*, which is a gavialoid species from the Maastrichtian (uppermost Cretaceous) Couche III level of the Moroccan phosphate series (Jouve et al., 2008). A third gavialoid species has also been described from this phosphate series, with *Maroccosuchus zennaroi* (Jonet & Wouters, 1977) present in the Ypresian Couche I level (Jouve et al., 2015), that is, the same level as NHMUK PV R36872. Although it has not been previously described, NHMUK PV R36872 was utilised by Rio and Mannion (2021) in their *Argochampsa krebsi* operational taxonomic unit. Given that NHMUK PV R36872 is from stratigraphically younger levels than previously described remains of *Argochampsa krebsi*, below we outline our reasoning for assigning it to this species.

Firstly, NHMUK PV R36872 possesses an elongate, narrow longirostrine snout which, despite missing the tip of the premaxilla, represents approximately 74% of the whole skull length (Figures, 2, 3, and 4). The snout shape of NHMUK PV R36872 is comparable to that of *Argochampsa* and *Ocepesuchus* (Hua & Jouve, 2004; Jouve et al., 2006, 2008), but contrasts with the mediolaterally wide and relatively short snout of *Maroccosuchus* (Jouve et al., 2015; Figure 5). Unfortunately, many of the proposed autapomorphies of *Argochampsa* pertain to the anteriormost part of the snout (Hua & Jouve, 2004; Jouve et al., 2006), which is damaged in NHMUK PV R36872, precluding their identification. Nevertheless, there are a number of proposed autapomorphies (Jouve et al., 2006) and other features from the remainder of the skull that can be evaluated.

**FIGURE 2:**
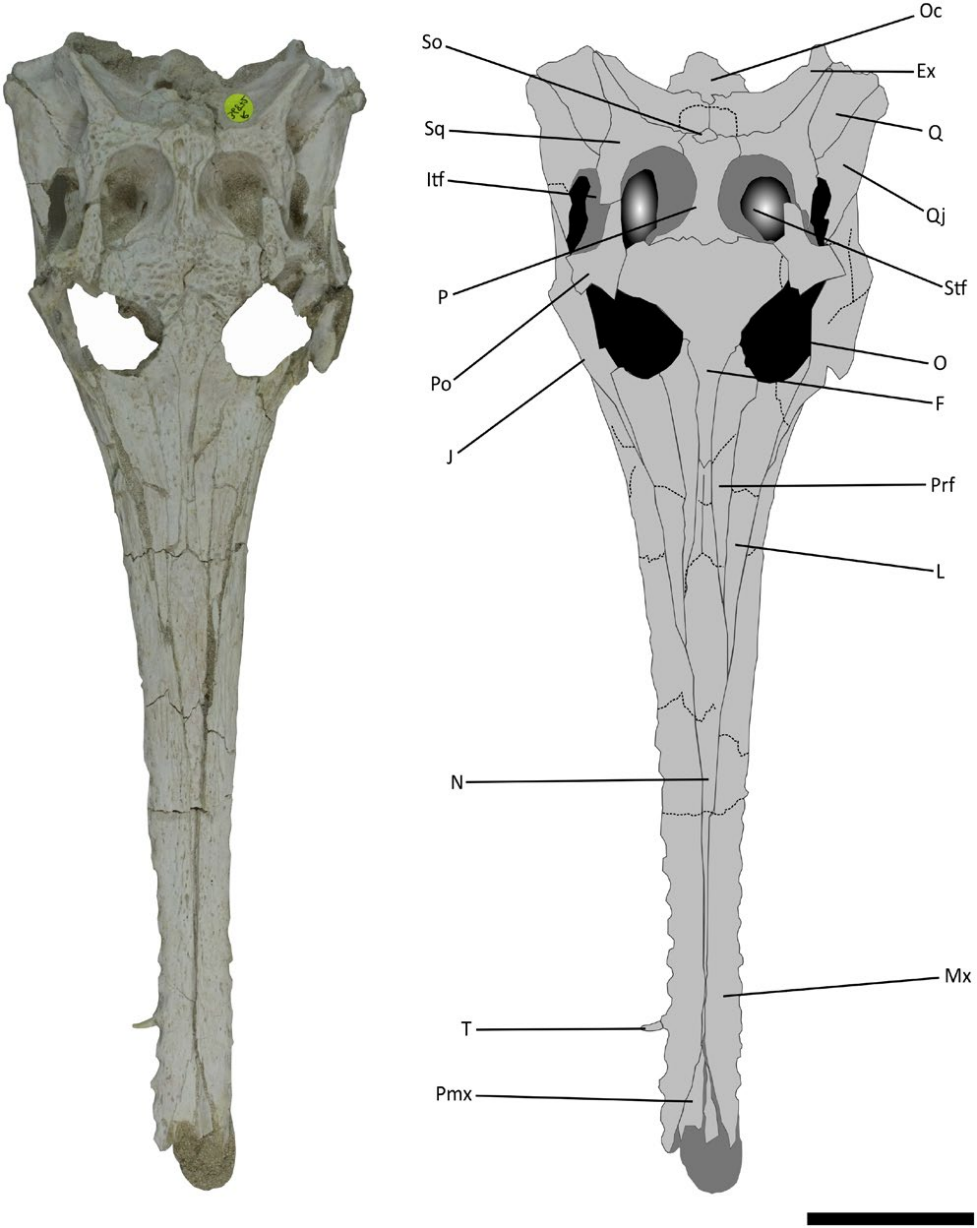
Skull of *Argochampsa krebsi* (NHMUK PV R36872) and its reconstruction in dorsal view. Ex, exoccipital; F, frontal; Itf, infratemporal fenestra; J, jugal; L, lacrimal; Mx, maxilla; N, nasal; O, orbit; Oc, occipital condyle; P, parietal; pmx; premaxilla; Po, postorbital; Prf, prefrontal; Q, quadrate; Qj, quadratojugal; So, supraoccipital; Sq, squamosal; Stf, supratemporal fenestra; T, tooth. Scale bar = 5 cm.

**FIGURE 3:**
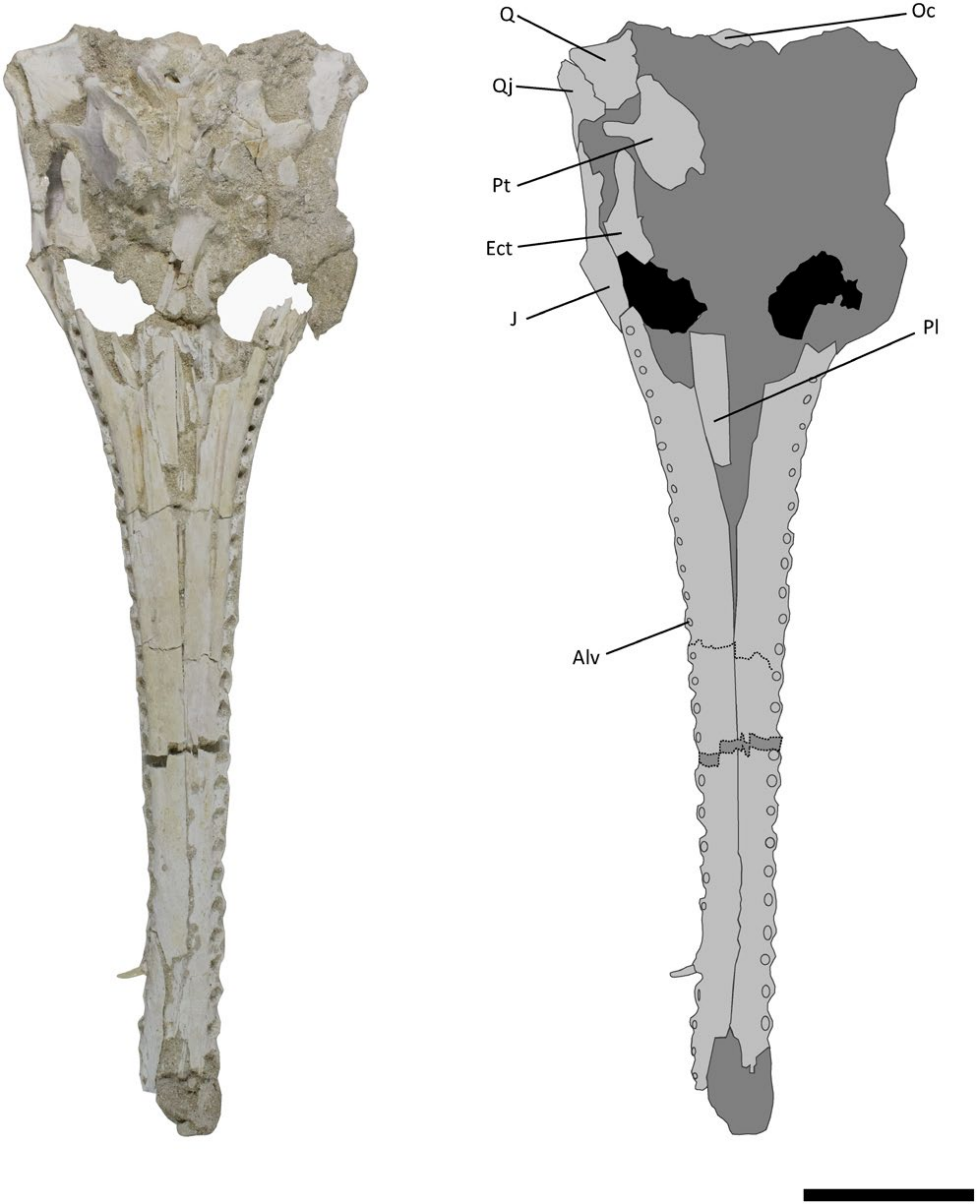
Skull of *Argochampsa krebsi* (NHMUK PV R36872) and its reconstruction in ventral view. Alv, alveolus; Ect, ectopterygoid; J, jugal; Oc, occipital condyle; Pl, palatine; Pt, pterygoid; Q, quadrate; Qj, quadratojugal. Scale bar = 5 cm.

**FIGURE 4:**
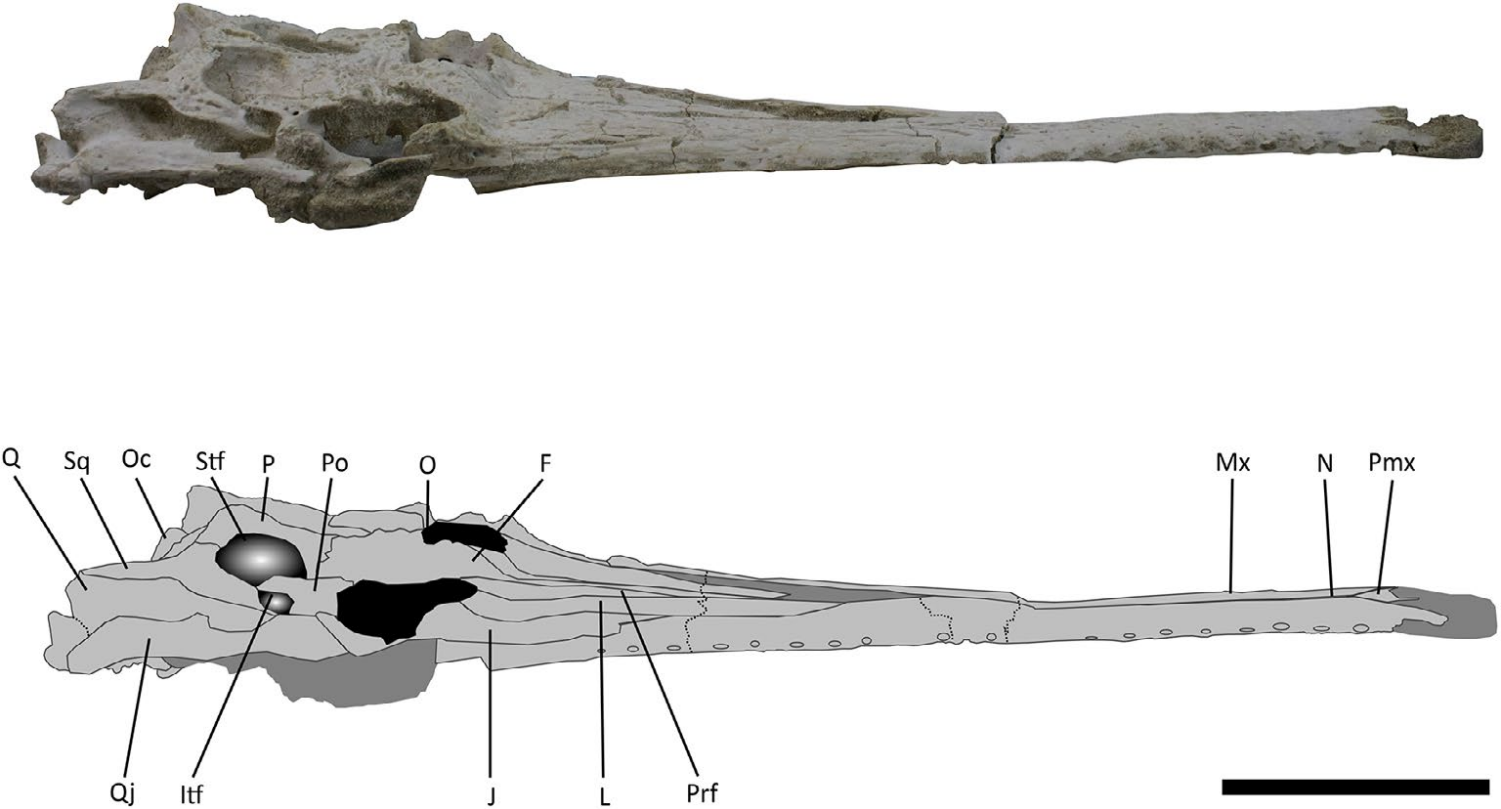
Skull of *Argochampsa krebsi* (NHMUK PV R36872) and its reconstruction in lateral view. Ect, ectopterygoid; F, frontal; Itf, infratemporal fenestra; J, jugal; L, lacrimal; Mx, maxilla; N, nasal; O, orbit; Oc, occipital condyle; P, parietal; pmx; premaxilla; Po, postorbital; Prf, prefrontal; Q, quadrate; Qj, quadratojugal; Sq, squamosal; Stf, supratemporal fenestra. Scale bar = 5 cm.

**FIGURE 5:**
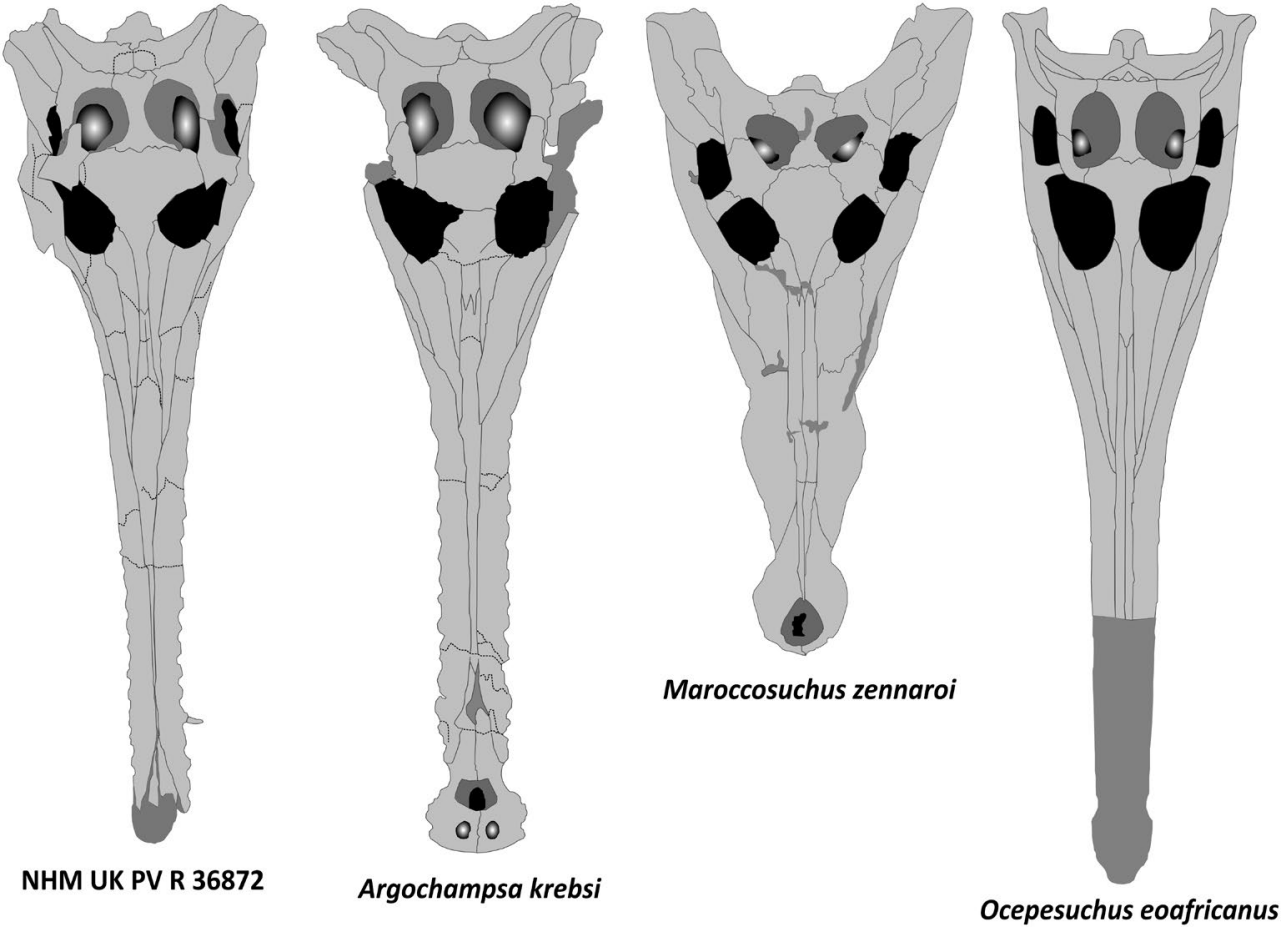
Line drawings of the skulls of NHMUK PV R36872, *Argochampsa krebsi* (OCP DEK‐GE 1201), *Maroccosuchus zennaroi* (MHNT.PAL.2006.80.11), and *Ocepesuchus eoafricanus* (OCP DEK‐GE 45).

**FIGURE 6:**
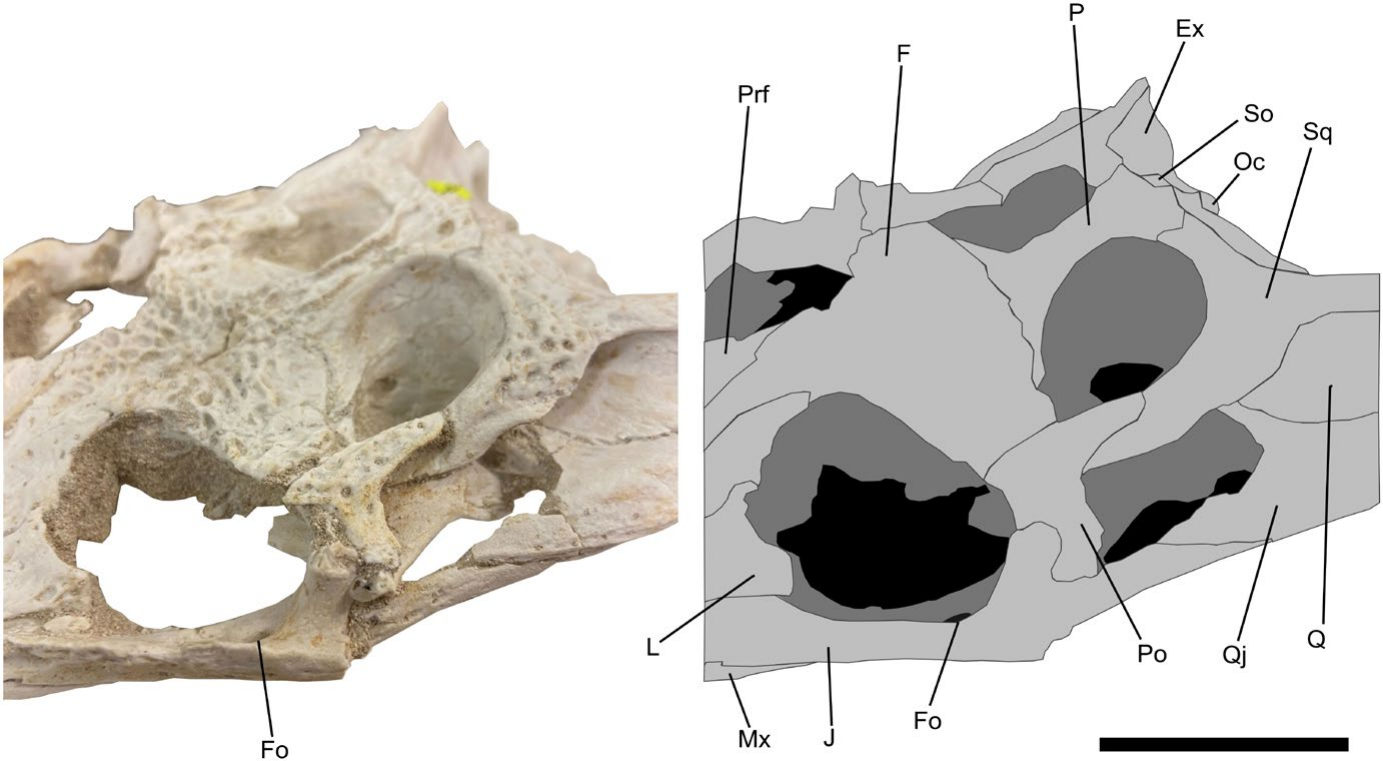
Skull of *Argochampsa krebsi* (NHMUK PV R36872) and its reconstruction in anterior oblique view. Ex, exoccipital; F, frontal; Fo, foramen; J, jugal; L, lacrimal; Mx, maxilla; Oc, occipital condyle; P, parietal; Po, postorbital; Prf, prefrontal; Q, quadrate; Qj, quadratojugal; So, supraoccipital; Sq, squamosal. Scale bar = 5 cm.

Like those of *Argochampsa* (Jouve et al., 2006), the nasals of NHMUK PV R36872 are fused, differing from the clearly distinguishable nasals of *Ocepesuchus* (Jouve et al., 2008). The depth of frontal penetration of the supratemporal fenestra in NHMUK PV R36872 is similar to that of *Argochampsa* (Hua & Jouve, 2004), whereas it is shallower in *Ocepesuchus* (Jouve et al., 2008). The interorbital space of NHMUK PV R36872 is much wider relative to the size of the orbits than that of *Ocepesuchus*, but this ratio is comparable to that of *Argochampsa* (Hua & Jouve, 2004; Jouve et al., 2006; Jouve et al., 2008; Figure 5). An external foramen can be observed between the maxilla and the jugal of NHMUK PV R36872. This foramen is absent in *Ocepesuchus*, and was identified as an autapomorphy of *Argochampsa* (Jouve et al., 2006; Jouve et al., 2008; Figures 3 and 6). As is the case in *Argochampsa* and *Ocepesuchus* (Hua & Jouve, 2004; Jouve et al., 2006), the posterolateral process of the squamosal of NHMUK PV R36872 is oriented posterolaterally. However, the orientation of this process is more similar to that of *Argochampsa*, with this process more laterally deflected in *Ocepesuchus*. The exoccipitals of NHMUK PV R36872 form a long, nearly horizontal plate, as is observed in *Argochampsa* (Jouve et al., 2006), rather than the shorter, completely horizontal plate seen in *Ocepesuchus* (Jouve et al., 2008). NHM UK PV R36872 does not exhibit a posterior process on its postorbital bar and its paroccipital processes are shaped as elongated narrow peaks that are posterolaterally oriented (Figure 4). These features were presented as autapomorphies for *Argochampsa* by Jouve et al. (2006).

In summary, NHMUK PV R36872 clearly differs from both *Maroccosuchus zennaroi* and *Ocepesuchus eoafricanus*, whereas it shares a combination of features that are otherwise known only in *Argochampsa krebsi*, to which we assign this specimen.

## 4 SYSTEMATIC PALAEONTOLOGY

Eusuchia Huxley, 1875.;

Crocodylia Gmelin, 1789;

Gavialoidea Hay, 1930;

Argochampsa Hua & Jouve, 2004;

Argochampsa krebsi Hua & Jouve, 2004.

4.1

**Holotype:** OCP DEK‐GE 1201—almost complete skull and mandibular fragments (see Hua & Jouve, 2004).

4.2

**Referred specimens:** Rhinopolis Collections, Phosphate 1 (Jouve et al., 2006)—nearly complete skull (lacking premaxillae), anterior portion of the mandible, the axis, one cervical vertebra, atlantal and axial ribs, one cervical rib, five vertebrae, proximal heads of the right and left humeri. OCP DEK‐GE 333 (Jouve et al., 2006)—well preserved skull. OCP DEK‐GE 1204 (Jouve et al., 2006)—poorly preserved skull that preserves the occipital area, parietal, postorbital, a fragment of the pterygoid, and a rostral fragment (lacking the anterior part from the suborbital fenestra to the premaxilla). NHMUK PV R36872—almost complete skull, lacking the anterior part of the rostrum (from the premaxilla). The skull is dorsoventrally crushed, and the ventral part of the skull is poorly preserved.

4.3

**Type locality and horizon:** Grand Daoui, Khouribga Province, Morocco; Couche II, upper Danian to lower Thanetian, Paleocene.

4.4

**Diagnosis:** Rostrum approximately 70% of the medial length of the skull; five premaxillary alveoli; 26 maxillary alveoli; premaxilla transversely broad and strongly bent downwards, with the first three alveoli forming a transverse row; diastema between the fourth and fifth premaxillary alveoli; nasals fused; no postorbital process on the postorbital bar; one external foramen between maxilla and jugal; frontal modestly penetrates supratemporal fenestra; paroccipital processes form two posterolaterally directed long narrow peaks; exoccipitals form a long nearly horizontal plate broadly visible in dorsal view; strong apophysis on the lateral margin of the odontoid process, continuous on the anterolateral margin of the centrum; neural spines of the posterior cervical vertebrae very low (Hua & Jouve, 2004; Jouve et al., 2006).

## 5 DESCRIPTION AND COMPARISONS

### 5.1 Brain endocast

**FIGURE 7:**
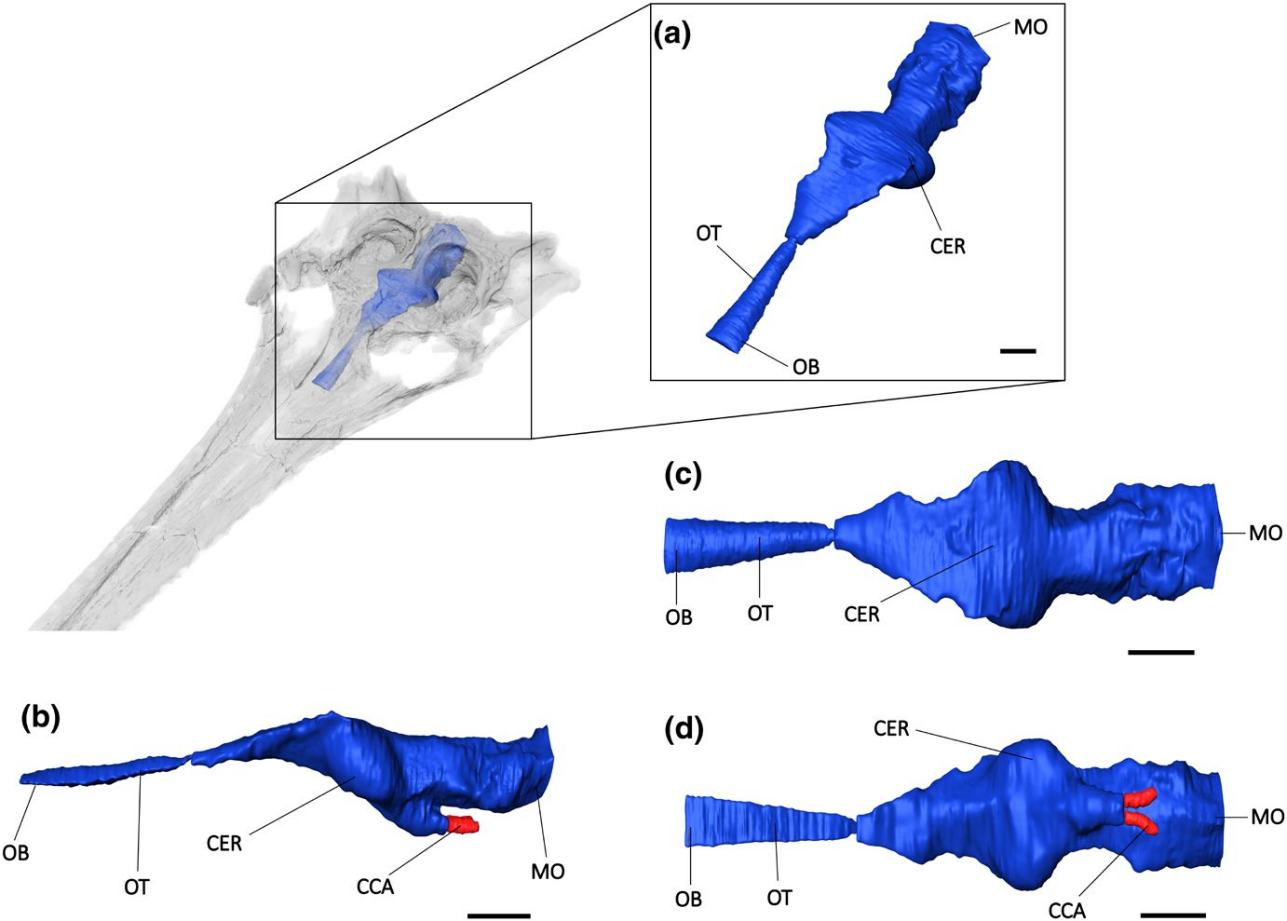
The brain endocast of *Argochampsa krebsi* (NHMUK PV R36872) in (a) anterior oblique, (b) lateral view, (c) dorsal and (d) ventral view. CER, cerebrum; CCA, cerebral carotid arteries; MO, medulla oblongata; OB, olfactory bulb; OT, olfactory tract; PIT, pituitary fossa. Scale bars = 10 mm.

The brain endocast of *Argochampsa krebsi* (NHMUK PV R36872) shows little curvature, similar to that of *Gavialis gangeticus* (Burke & Mannion, 2023; Table 1; Figure 7). Comparable to other eusuchians (Burke & Mannion, 2023; Serrano‐Martínez et al., 2021), the brain endocast of *Argochampsa* displays a sigmoidal morphology in lateral view. The protrusion of the dorsal longitudinal sinus is less pronounced in *Argochampsa* than it is for *Gavialis gangeticus* and *Eosuchus lerichei* (Dollo, 1907) (Burke & Mannion, 2023, Burke et al., 2024a; Figure 7(b)). There is a lack of osteological division of the olfactory bulb from the olfactory tract in *Argochampsa*.

In comparison with the rest of the brain endocast, *Argochampsa* has a laterally expansive and bulbous cerebrum (Figure 4). The ratio of the mediolateral cerebrum width to skull width in lateral view is greater than that of both *Gavialis gangeticus* and *Tomistoma schlegelii* (Table 2). As is also the case in *Gavialis gangeticus*, *Sutekhsuchus dowsoni* (Fourtau, 1920), and *Eosuchus lerichei*, the largest expansion in dorsal view occurs at the posterior end of the cerebrum (Burke & Mannion, 2023; Burke et al., 2024a; Figure 7). The pituitary of *Argochampsa* is much shorter anteroposteriorly relative to that of extant gavialoid species, although it has a similar mediolateral width as those two species (Table 1). A posterolateral expansion of the pituitary to house the cerebral carotid artery can be seen in *Argochampsa*, as is also the case in *Gavialis*, *Tomistoma*, and *Eosuchus lerichei* (Burke et al., 2024a; Burke & Mannion, 2023). The smaller size of the pituitary fossa relative to the endocast length in *Argochampsa* suggests that it had a slightly reduced pituitary gland.

*Argochampsa* has the same cerebrum width to skull width ratio as *Sutekhsuchus*, suggesting that the two species had very similar behavioural complexities, closer to that of *Gavialis* than any other extant crocodylian (Pierce et al., 2017; Burke & Mannion, 2023; Table 2).

### 5.2 Endosseous labyrinth

**FIGURE 8:**
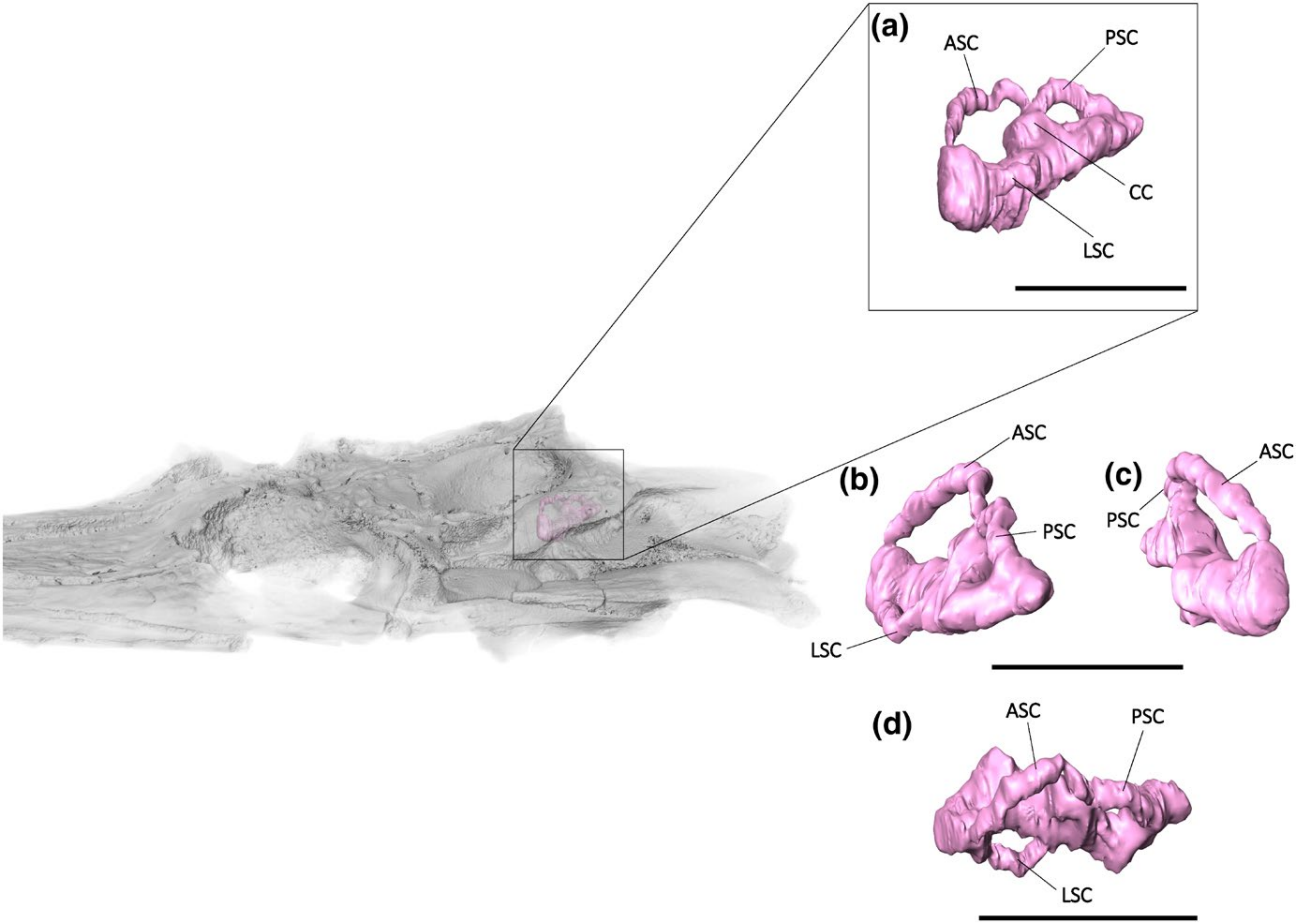
The endosseous labyrinth of *Argochampsa krebsi* (NHMUK PV R36872) in (a) lateral, (b) posterior, (c) anterior and (d) ventral view. ASC, anterior semicircular canal; CC, common crux; LSC, lateral semicircular canal; PSC, posterior semicircular canal. Scale bars = 10 mm.

The region of the skull housing the endosseous labyrinth is poorly preserved in NHMUK PV R36872 because of deformation. Despite this, the overall shape, excluding the cochlear duct, can be reconstructed. Like most archosaurs (Brusatte et al., 2016), the anterior semi‐circular canal of *Argochampsa* is greater than four times the size of the posterior semi‐circular canal (Figure 8; Table 2). The semi‐circular canals of *Argochampsa* are thicker than those of *Gavialis* and *Sutekhsuchus*, comparable to those of *Eosuchus lerichei* (Burke et al., 2024a), although they are thinner than the canals of most metriorhynchid thalattosuchians (Schwab et al., 2020). In *Argochampsa*, the cochlear duct is only slightly expanded mediolaterally, with the length of the endosseous cochlea relative to the height of the endosseous labyrinth being slightly lower than that of both *Gavialis gangeticus* and *Tomistoma schlegelii* (Figure 8; Table 1). However, the apparent short length of the cochlea of *Argochampsa* is most likely caused by the poor preservation of NHMUK PV R36872.

### 5.3 Nasal cavity and associated structures

**FIGURE 9:**
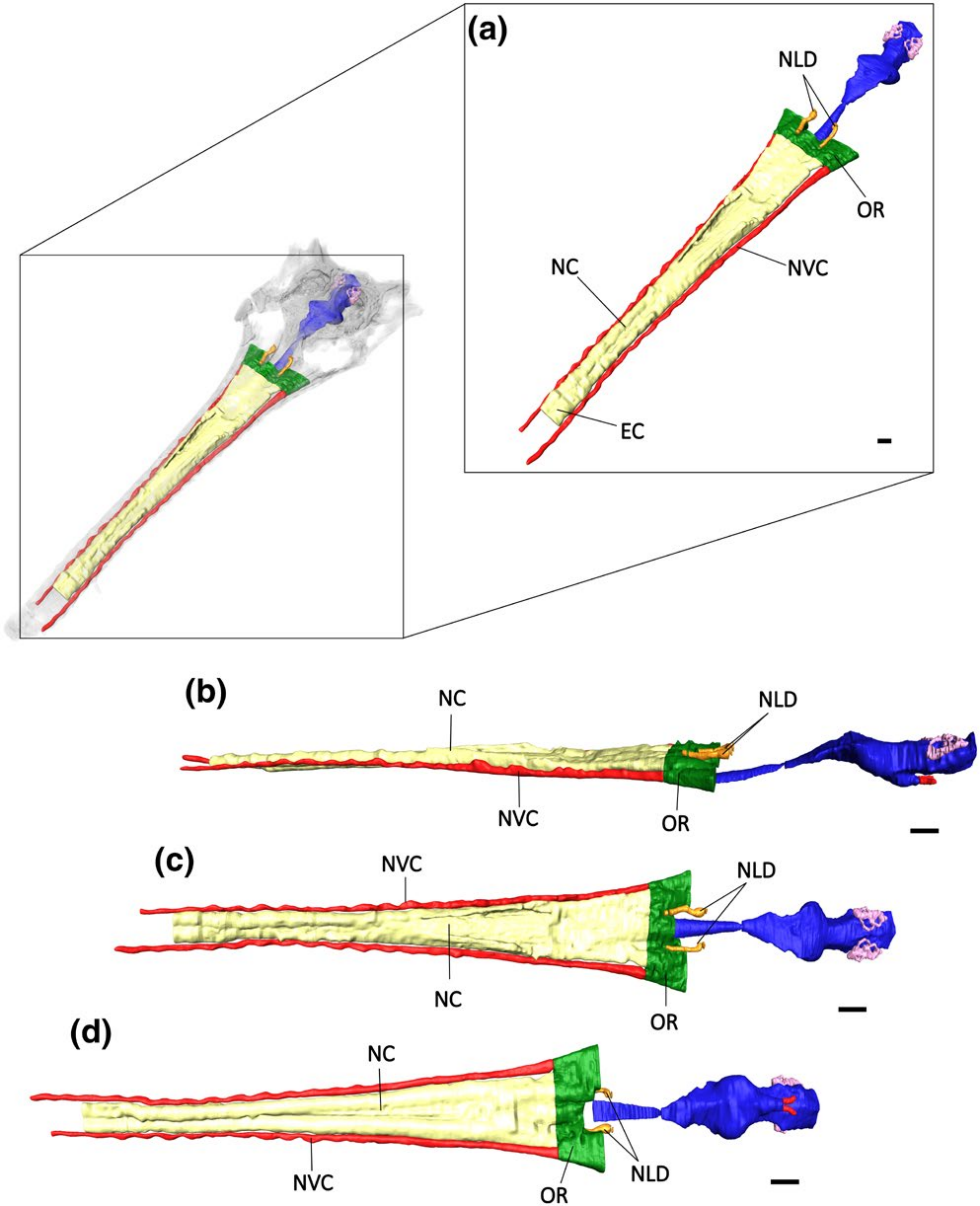
The nasal endocast of *Argochampsa krebsi* (NHMUK PV R36872) in (a) anterior oblique, (b) lateral, (c) dorsal, and (d) ventral view. EC, external choana; NC, nasal cavity; NLD, nasolacrimal duct; NVC, neurovascular canals; OR, olfactory region. Scale bars = 10 mm.

The nasal cavity of *Argochampsa* (NHMUK PV R36872) extends posteriorly from the maxillae to the prefrontals (Figure 9). As a result of the dorsoventral compression of NHMUK PV R36872, the external choana could not be segmented. The region between the prefrontals and the basicranium is also poorly preserved in NHMUK PV R36872 (Figure 9). Both the ventral and dorsal surfaces of the nasal cavity of *Argochampsa* are characterised by a pronounced longitudinal groove that mirrors the anteroposterior extension of the nasal bone.

The nasal cavity along the maxillae mirrors the shape of the rostrum, widening laterally anterior to the endocranium to form the olfactory region (Figure 9). Unlike the more lateral expansion observed in *Eosuchus lerichei* (Burke et al., 2024a), the olfactory region in *Argochampsa* exhibits a dorsolateral expansion.

The internal surfaces of the prefrontals and lacrimals of *Argochampsa* are characterised by concave depressions. Similar features in some gavialoids and metriorhynchoid thalattosuchians have been proposed to be osteological correlates of nasal salt glands (see Discussion Burke et al., 2024a; Cowgill et al., 2023). The olfactory bulb does not connect to the olfactory region in NHMUK PV R36872. However, this could be due to the fragmentation of the specimen which caused the displacement of the olfactory bulb and olfactory tract.

The nasolacrimal ducts of *Argochampsa* are relatively straight and run anteroposteriorly parallel to each other, similar to *Eosuchus lerichei* and *Sutekhsuchus* (Burke et al., 2024a; Burke & Mannion, 2023). Unlike *Eosuchus lerichei*, *Gavialis*, and *Sutekhsuchus* (Burke et al., 2024a; Burke & Mannion, 2023), the nasolacrimal ducts in *Argochampsa* do not fully extend posteriorly from the orbits to the anterior end of the dorsal surface of the olfactory region (Figure 9(c)). However, this is likely due to the poor preservation of NHMUK PV R36872.

The neurovascular canals on the lateral surfaces of the nasal cavity run anteroposteriorly from the olfactory region to the premaxilla in *Argochampsa* (Figure 9). Unlike other gavialoids, such as *Eosuchus lerichei* and *Gavialis* (Burke et al., 2024a; Burke & Mannion, 2023), these canals do not converge on the dorsal surface of the nasal cavity although this has likely been affected by the poor preservation of the rostral extremity of NHMUK PV R36872.

## 6 DISCUSSION

The morphology of the endosseous labyrinth of crocodylomorphs has been linked to the environment these species inhabited (Schwab et al., 2020; Ristevski, 2022). The thick semi‐circular canals of metriorhynchid thalattosuchians reflect their pelagic ecology, whereas fully terrestrial species have thinner canals (Schwab et al., 2020). The gavialoids *Gavialis* and *Sutekhsuchus* show an intermediate morphology (Burke & Mannion, 2023), whereas *Argochampsa* and *Eosuchus lerichei* are characterised by semi‐circular canals that are thicker than those of the aforementioned gavialoids, but thinner than those of pelagic metriorhynchids. This suggests that *Argochampsa* (and *Eosuchus*) inhabited pelagic environments more frequently than *Gavialis* and *Sutekhsuchus*, although were not obligately pelagic.

**FIGURE 10:**
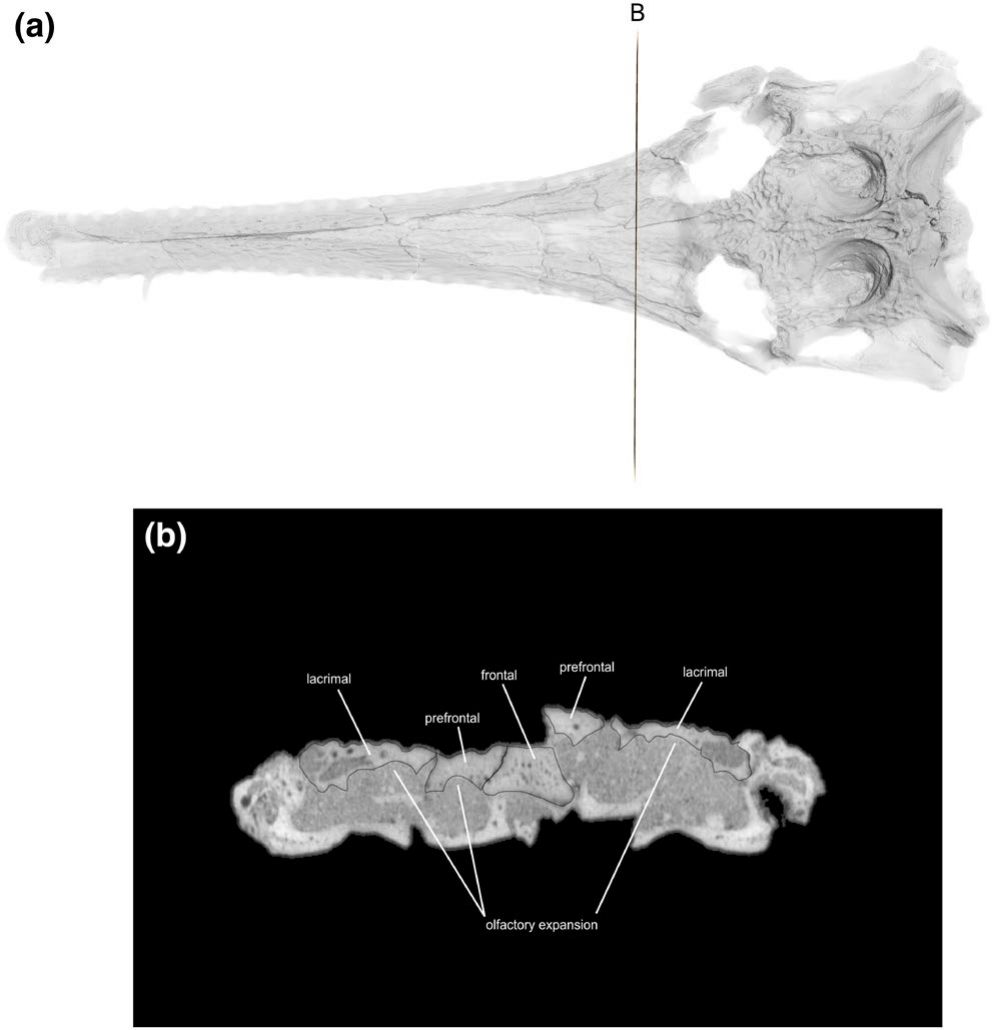
(a) The skull rendering of *Argochampsa krebsi* (NHMUK PV R36872) in dorsal view, with (b) showing the concave depressions on the internal surface of the prefrontal and lacrimal bones indicating the olfactory expansions and salt glands.

**FIGURE 11:**
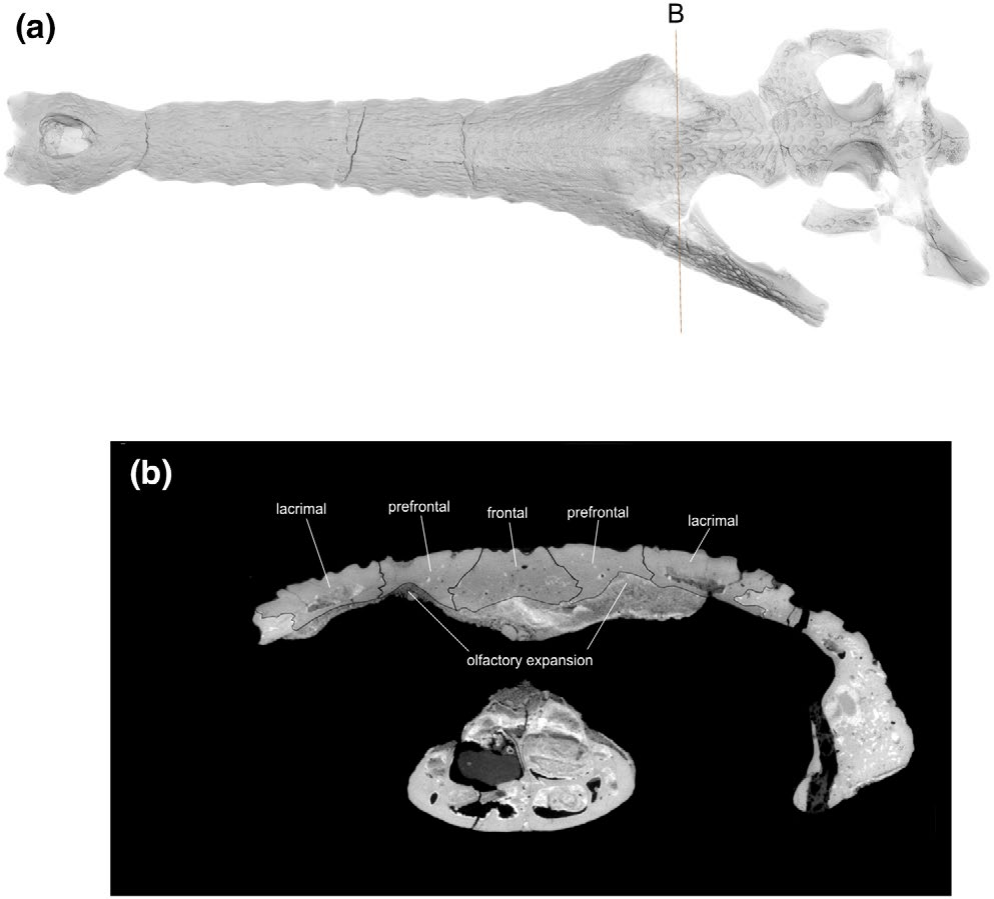
(a) The skull rendering of *Sutekhsuchus dowsoni* (NHMUK PV R4769) in dorsal view, with (b) showing the concave depressions on the internal surface of the prefrontal and lacrimal bones indicating the olfactory expansions and salt glands.

The hypothesis that *Argochampsa* regularly inhabited pelagic environments is further supported by the presence of concave depressions on the internal surface of the lacrimals and prefrontals (Figure 10). The natural endocasts of some pelagic metriorhynchid thalattosuchians indicate that these depressions correspond to nasal salt glands (Cowgill et al., 2023; Fernández & Gasparini, 2000; Herrera et al., 2013), and they have therefore been interpreted as osteological correlates for salt glands in numerous additional metriorhynchoids as well as some extinct gavialoid species (Burke et al., 2024a). Despite the dorsoventral compression in NHMUK PV R36872, the depressions observed on the internal surface of the lacrimals and prefrontals provide support for the hypothesis that any large expansion ventral to the lacrimals and prefrontals are salt glands (Pierce et al., 2017; Figure 10a,b). The salt glands observed in metriorhynchoids such as *Cricosaurus araucanensis* (Gasparini & Dellapé, 1976), *Pelagosaurus typus* (Bronn, 1841), and *Eoneustes gaudryi* (Collot, 1905) were inferred from similar concave depressions on the internal surface of the lacrimals and prefrontals, although they are located more laterally in the posterior olfactory region than those of extinct gavialoids, connecting to the preorbital fenestrae (see Cowgill et al., 2023, Figures 4 and 5; Fernández & Herrera, 2021, Figure 3). The latter opening in metriorhynchoids, a feature absent in gavialoids, has been suggested to contain a conduit running from the olfactory region that played a role in the drainage of the salt ducts (Cowgill et al., 2023; Fernández & Herrera, 2009). The natural endocast of *Dakosaurus andiniensis* (Vignaud & Gasparini, 1996) shows that its salt glands are located more medially than in other metriorhynchoids (Fernández & Herrera, 2021), similar to the inferred position of salt glands in *Argochampsa* (Figure 10) and other fossil gavialoids (Burke et al., 2024a). The depressions on the internal surface of the prefrontals and lacrimals of NHMUK PV R36872 are also more anteroposteriorly oriented than those of metriorhynchoids and are more similar to those of *Eosuchus lerichei* and *Sutekhsuchus* (Figure 11(a)), reflecting the orientation of the salt glands seen in extant marine iguanas (Dunson, 1969; Burke et al., 2024a; Burke et al., 2024b; Figure 10). Hence, *Argochampsa krebsi*, *Eosuchus lerichei*, *Portugalosuchus azenhae* (Mateus et al., 2018), *Sutkehsuchus dowsoni* and metriorhynchoids all exhibit the same osteological correlate for salt glands, although the location and orientation of the salt glands differ (Burke et al., 2024a; Cowgill et al., 2023; Puértolas‐Pascual et al., 2023).

**FIGURE 12:**
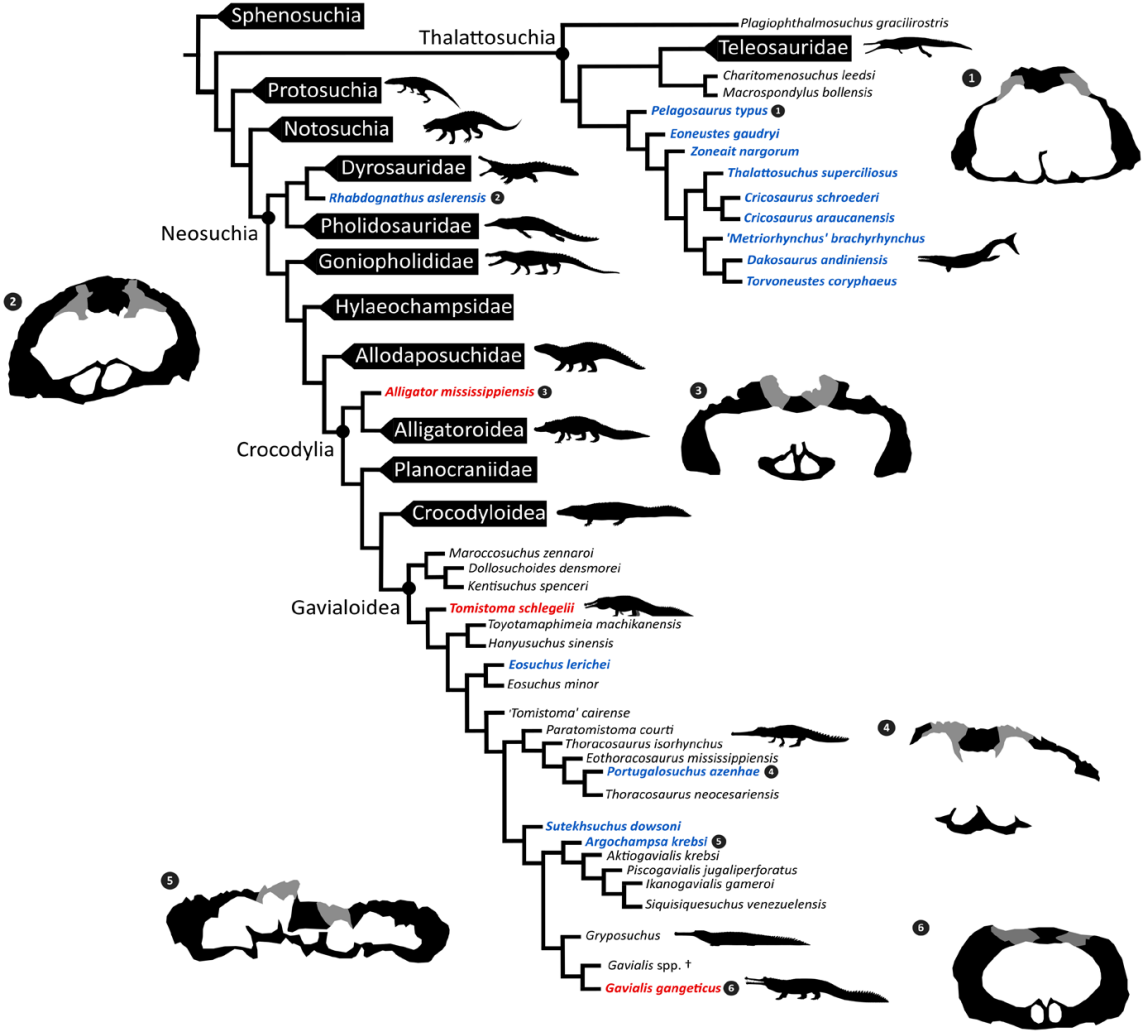
Phylogenetic tree showing the evolutionary relationships of Crocodyliformes, highlighting species with osteological correlates of salt glands, adapted from trees by Wilberg et al. (2019); Rio and Mannion (2021); Burke et al. (2024a, 2024b); Young et al. (2024); Forêt et al. (2024). Taxa in blue = evidence of osteological correlates for salt glands; red = osteological correlates absent, black = unknown. Numbers 1–6 correspond to the olfactory regions showing placement of the concave depressions of representative species.

Amongst non‐thalattosuchian and non‐gavialoid pelagic crocodyliforms, there are currently no digital reconstructions of the endocranial anatomy of pholidosaurids: there is only an interpretive drawing of the reconstructed endocranial anatomy of pholidosaurids (Figure 12), a lateral view of brain endocast of *Pholidosaurus meyeri* that does not provide information regarding the possible presence of salt glands in this clade (Hopson, 1979, Figure 10). There is only one reconstruction of the endocranial anatomy of a dyrosaurid, the Paleocene northwest African species *Rhabdognathus aslerensis* (Jouve, 2007) (Erb & Turner, 2021). Although this was not described, this species appears to also possess concave depressions in the prefrontals (Erb & Turner, 2021, Figure 3).

Thus, Dyrosauridae, Gavialoidea, and Metriorhynchoidea are currently the only known clades that exhibit osteological correlates for nasal salt glands (Cowgill et al., 2021; Burke et al., 2024a). Although this likely represents a convergent ecological adaptation, rather than an apomorphic loss in some lineages, much of the crocodyliform evolutionary tree remains unsampled for these osteological correlates (Figure 12).

The presence of salt glands in early gavialoid species supports the hypothesis that gavialoid lineages were capable of transoceanic dispersal (Burke et al., 2024a; Delfino et al., 2005). Furthermore, the recent phylogeny of Burke et al. (2024a) provides support for the hypothesis that the capacity for salt excretion is an ancestral trait of Gavialoidea. Nevertheless, information on additional species is needed to test this. Analysis of the endocranial anatomy of *Aktiogavialis* (Vélez‐Juarbe et al., 2007), a gavialoid from Central and northern South America that is closely related to *Argochampsa* (Salas‐Gismondi et al., 2019; Vélez‐Juarbe et al., 2007), could help determine whether nasal salt glands evolved independently in *Argochampsa* and *Eosuchus lerichei*.

## 7 CONCLUSIONS

We present a reconstruction of the endocranial anatomy of the early Paleogene North African gavialoid crocodylian *Argochampsa krebsi* using CT‐scan data. The internal surface of the prefrontals and lacrimals of *Argochampsa* are characterised by concave depressions, indicating that that this species possesses osteological correlates for nasal salt glands. This signifies that *Argochampsa* most likely frequented pelagic environments, providing further anatomical evidence that extinct gavialoids were capable of transoceanic dispersal. Appearing in Dyrosauridae, Thalattosuchia, and Gavialoidea, we hypothesise that salt glands have evolved convergently across Crocodyliformes, and our study highlights gaps within this clade's evolutionary history which should be the focus of future study.

## Data Availability

The data that support the findings of this study are openly available in Morphosource.
